# Immunologic aspects of asthma: from molecular mechanisms to disease pathophysiology and clinical translation

**DOI:** 10.3389/fimmu.2024.1478624

**Published:** 2024-10-08

**Authors:** Cong Xie, Jingyan Yang, Aman Gul, Yifan Li, Rui Zhang, Maimaititusun Yalikun, Xiaotong Lv, Yuhan Lin, Qingli Luo, Huijuan Gao

**Affiliations:** ^1^ Department of Endocrinology and Clinical Immunology, Yuquan Hospital, School of Clinical Medicine, Tsinghua University, Beijing, China; ^2^ Department of Integrative Medicine, Huashan Hospital Affiliated to Fudan University, Fudan Institutes of Integrative Medicine, Fudan University Shanghai Medical College, Shanghai, China; ^3^ The Third Affiliated Hospital, Beijing University of Chinese Medicine, Beijing, China; ^4^ Department of Respiratory Medicine, Uyghur Medicines Hospital of Xinjiang Uyghur Autonomous Region, Urumqi, China; ^5^ College of Life Science and Technology, Xinjiang University, Urumqi, China; ^6^ Department of Pulmonary and Critical Care Medicine, Shenzhen Hospital of Guangzhou University of Chinese Medicine (Futian), Shenzhen, China; ^7^ Department of Cardiology, The Second Affiliated Hospital of Tianjin University of Traditional Chinese Medicine, Tianjin, China; ^8^ Dongzhimen Hospital, Beijing University of Chinese Medicine, Beijing, China

**Keywords:** lung, asthma, immunity, inflammation, allergy, T_H_2 cytokines, immunotherapy

## Abstract

In the present review, we focused on recent translational and clinical discoveries in asthma immunology, facilitating phenotyping and stratified or personalized interventions for patients with this condition. The immune processes behind chronic inflammation in asthma exhibit marked heterogeneity, with diverse phenotypes defining discernible features and endotypes illuminating the underlying molecular mechanisms. In particular, two primary endotypes of asthma have been identified: “type 2-high,” characterized by increased eosinophil levels in the airways and sputum of patients, and “type 2-low,” distinguished by increased neutrophils or a pauci-granulocytic profile. Our review encompasses significant advances in both innate and adaptive immunities, with emphasis on the key cellular and molecular mediators, and delves into innovative biological and targeted therapies for all the asthma endotypes. Recognizing that the immunopathology of asthma is dynamic and continuous, exhibiting spatial and temporal variabilities, is the central theme of this review. This complexity is underscored through the innumerable interactions involved, rather than being driven by a single predominant factor. Integrated efforts to improve our understanding of the pathophysiological characteristics of asthma indicate a trend toward an approach based on disease biology, encompassing the combined examination of the clinical, cellular, and molecular dimensions of the disease to more accurately correlate clinical traits with specific disease mechanisms.

## 1 Introduction

Asthma is a familiar, chronic, noncommunicable lung disease affecting approximately 300 million people worldwide (1), including 45.7 million adults in China (2). The prevalence of asthma considerably varies across different countries and regions; the prevalence is higher in urban areas and individuals with some risk factors, including allergies, smoking, and air pollution exposure. Although asthma incidence appears to be stabilizing after decades of rapid growth in many developed countries, its prevalence is increasing rapidly in low- and middle-income countries. This increase in prevalence may be owing to the worsening of fossil fuel pollution and the adoption of Westernized lifestyles. Furthermore, the absence of accurate diagnosis and standardized treatments in these developing countries increases the asthma burden on patients, their families, and society as a whole (3).

An expiratory airflow limitation is the primary feature of asthma; however, this limitation is generally reversible but related to airway lumen diameter narrowing. The narrowing occurs because of chronic inflammation in the walls of the airway, which is marked by the infiltration and activation of different immune cells, including eosinophils, neutrophils, lymphocytes, dendritic cells (DCs), innate lymphoid cells (ILCs), and mast cells, inducing processes such as bronchial hyperresponsiveness, mucus hypersecretion, and airway remodeling.

The clinical traits of asthma, i.e., dyspnea, coughing, wheezing, chest tightness, loss of lung function, exacerbation tendency, and asthma severity, suggest that the disease encompasses distinct underlying mechanisms, in which structural and immune cells interact to manifest the pathogenetic features of asthma. However, the relative contribution of these features may differ among patients with asthma, coupled with remarkable differences in genetic variations and environmental exposure; this results in significant heterogeneity in clinical manifestations and inflammatory biomarker expression.

Asthma has different clinical characteristics (“phenotypes”) and underlying causative mechanisms (“endotypes”) (4). Historically, clinicians have categorized asthma into two phenotypes: intrinsic (nonallergic) and extrinsic (allergic) (5). The primary difference between these two phenotypes is that allergic asthma generally occurs during childhood, whereas nonallergic asthma usually begins in adulthood. Allergic asthma typically manifests as acute episodes with increased airway responsiveness after allergen stimulation; it is more responsive to inhaled corticosteroids (ICSs) compared with nonallergic asthma (6). More recently, various clinical parameters, including onset age, condition severity and duration, frequency of acute exacerbation, impairment in respiratory function, level of symptom control, biomarkers, and treatment response, including potential hormone resistance, have been utilized to classify the phenotypes of asthma.

Some of the most common phenotypes, including allergic asthma, nonallergic asthma, adult-onset (late-onset) asthma, asthma with persistent airflow limitation, and obesity-associated asthma, have been listed in the updated 2023 and 2024 Global Initiative for Asthma (GINA) guidelines (7). Simultaneously, researchers in the field of basic medical sciences, particularly immunologists using murine models of allergic asthma and/or inflammation, have confirmed the pivotal role of the elements of the T helper (T_H_2) immune pathway in exacerbating inflammation and airway hyperreactivity (8, 9). T_H_2 cells are involved in the generation of cytokines that induce the different essential characteristics of asthma, including tissue eosinophilia (interleukin [IL]-5), bronchial hyperresponsiveness (IL-13), and goblet cell metaplasia (IL-4 and IL-13) (10). Recent studies have extended this understanding and suggested that apart from T_H_2 cells, other innate immune cells, including mast cells, basophils, group 2 ILCs, IL-4- and/or IL-13-activated macrophages (“M2”), and a small portion of IL-4-secreting natural killer (NK)/NKT cells, also contribute to T_H_2 cell induced cytokine production in asthma; as a result, the terminology has gradually shifted from “T_H_2 cell-high” to “type 2-high” asthma (11). However, this “type 2-high” profile, primarily characterized by eosinophilia, is only observed in roughly 50% of patients with asthma (12). The remaining patients, categorized as “type 2-low” asthma, without eosinophilia, exhibit distinct immune features, including airway neutrophilia, obesity-associated systemic inflammation, or minimal immune activation signs in some cases (13).

In patients with asthma, there is specific chronic inflammation in the lower airway mucosa. Although the major cellular components associated with this inflammation type have been ascertained, the interplay between the inflammatory cells in different spatial and temporal dimensions remains unclear (14); furthermore, it is not known how this inflammation translates into asthma symptoms. Similar to other atopic diseases, asthma pathogenesis involves several factors, including genetic predisposition, the airway initiation of specific IgE (sIgE) to respiratory allergens, and an overactive immune system that produces excessive amounts of inflammatory mediators. To date, the acute inflammatory changes observed in asthma have garnered considerable attention; in this chronic condition, inflammation persists for many years in most patients. Superimposed on this chronic inflammatory state are acute inflammatory episodes, which correspond to exacerbations of asthma.

Moving from patients to animal or cellular models and back represents an iterative process by which we can elucidate the intricate pathophysiology of asthma. Herein, we focus on the underlying immunological aspects of asthma in the context of recent insights into its extraordinary heterogeneity by summarizing the findings from human studies on particular pathways along with rigorous basic experimentation that has collected a surplus of molecular details.

## 2 Pro-inflammatory and anti-inflammatory arms of the immune landscape in asthma

Under the guidance of locally released chemokines, many inflammatory cells are recruited to the lungs from the bloodstream; these cells exert functional properties for asthma development. Furthermore, airway structural cells, including epithelial cells, fibroblasts, and airway smooth muscle cells (SMCs), are essential inflammatory mediator sources that actively participate in the inflammatory process. In individuals with asthma, both innate (mast cells, DCs, eosinophils, neutrophils, basophils, ILCs, monocytes, and macrophages) and adaptive (T and B lymphocytes) immunities are involved in the inflammatory cell profile (15).

A landmark study conducted in the mid-1980s reported the classical CD4^+^T lymphocyte subsets (T_H_1 and T_H_2 cells) (16); since then, it is well-known that T_H_2 cells orchestrate eosinophilic airway inflammation by producing abundant amounts of IL-4, IL-5, and IL-13 (17). IL-4 is required for allergic sensitization and IgE class switching, IL-5 is warranted for eosinophil survival, IL-13 exerts multifunctional effects in the lungs, including a vital role in controlling mucus production, goblet cell metaplasia, bronchial hyperresponsiveness, and airway remodeling (18). In contrast, T_H_1 cells release IL-2, interferon (IFN)-γ, and tumor necrosis factor (TNF)-α, possibly conferring a protective role in asthma because they can directly antagonize pathologic T_H_2 responses to control eosinophilic inflammation (19). To support this, IL-12, a pro-T_H_1 cell cytokine, administration in mice suppresses antigen-induced airway hyperresponsiveness and inflammation by producing IFN-γ via T_H_1 cells (20, 21). However, recent studies on the phenotype of type 2-low asthma have demonstrated the dominance of IFN-γ^+^T_H_1 cells in severe disease forms, which is potentially associated with corticosteroid refractoriness (22, 23).

In addition to T_H_2 cells, T_H_17 cells and their produced cytokine IL-17A are prominent and extensively studied in the context of asthma, particularly in severe, steroid-resistant cases (24). These T_H_17-derived cytokines, including IL-17 and IL-22, are related to increased neutrophil recruitment in the airways (25). To this end, neutrophil extracellular traps and cytoplasts further promote T_H_17 polarization and neutrophilic inflammation in severe asthma (26). However, the precise roles of T_H_17 cells and IL-17 in mouse asthma models remain unknown primarily because IL-17 may play dual regulatory roles: it plays a protective role in the challenge stage but worsens asthma under other conditions (27, 28). In chronic asthma models, IL-17A induces the proliferation of fibroblasts (29), inhibits the anti-inflammatory effects of regulatory T cells (Tregs) (30), and directly contracts bronchial SMCs (31).

Increasing evidence in animals indicates that a major hallmark of several autoimmune disorders, including asthma, is functional defects in Tregs (32). In a broader perspective, as a diverse population, Tregs comprise CD4^+^CD25^+^ forkhead box (Fox)p3^+^ natural and inducible Tregs, IL-10-producing Tr1 cells, transforming growth factor (TGF)-β-producing T_H_3 cells, and other minor subsets with suppressive functions, including CD4^−^CD8^−^ T and γδT cells (33). In children with asthma, both CD4^+^CD25^hi^ Tregs and Foxp3 mRNA expression decrease in the peripheral blood and bronchoalveolar lavage fluid (BALF); this phenomenon can be reversed following treatment with inhaled glucocorticoids (34). Recently, a study has revealed that numerical and functional deficiencies in Tregs may increase the asthma risk in children and young adults; however, the association between Tregs and the risk or severity of asthma in the elderly may be weaker (35).

The discovery of a distinct cohort of IL-9-secreting CD4^+^T cells, called T_H_9 cells, has enhanced the intricacies of T cell subsets. These cells are produced in response to IL-4 and TGF-β (36). These T_H_9 cells facilitate the binding of the transcription factors PU.1 and interferon-regulatory factor (IRF)-4 to the *Il9* promoter (37). Furthermore, IL-25 (i.e., IL-17E) enhances IL-9 secretion from T_H_9 cells (38). IL-9 promotes allergic responses, including IgE production and eosinophilia (39). In allergic inflammation experimental models, mast cell accumulation is IL-9-dependent (40); however, lung-infiltrating mast cells and protease expression in mast cells were significantly decreased in mice with PU.1 deficiency (41). Subsequent studies have demonstrated that the deletion of a regulatory region in the *Il9* locus, which is vital for initiating the IL-9 expression and T_H_9 cell maturation, effectively alleviates allergic lung inflammation (42, 43). Several clinical trials involving a humanized anti-IL-9 monoclonal antibody (mAb), MEDI-528, have been successfully completed in individuals with asthma, demonstrating some degree of efficacy (44, 45).

At present, studies suggest that alveolar macrophages possess comprehensive immunoregulatory capabilities in asthma, beyond those of a pathogenic barrier to lung tissues (46). Based on the stimulation type, surface markers, pattern of secreted cytokines, and functional characteristics, two main polarized macrophage subpopulations have been identified: “M1” macrophages, which are classically activated, and “M2” macrophages, which are alternatively activated (47). Although controversial, some studies have demonstrated that M2 macrophages express T_H_2-associated cytokines (IL-4 and IL-13) and TGF-β, which participate in type 2 inflammation and airway remodeling in allergic asthma (48). However, M2 macrophages release high levels of IL-10 and TGF-β, playing roles in inflammation resolution, wound repair, and homeostasis maintenance, further complicating the precise function of M2 macrophages (49).

**Figure 1:**
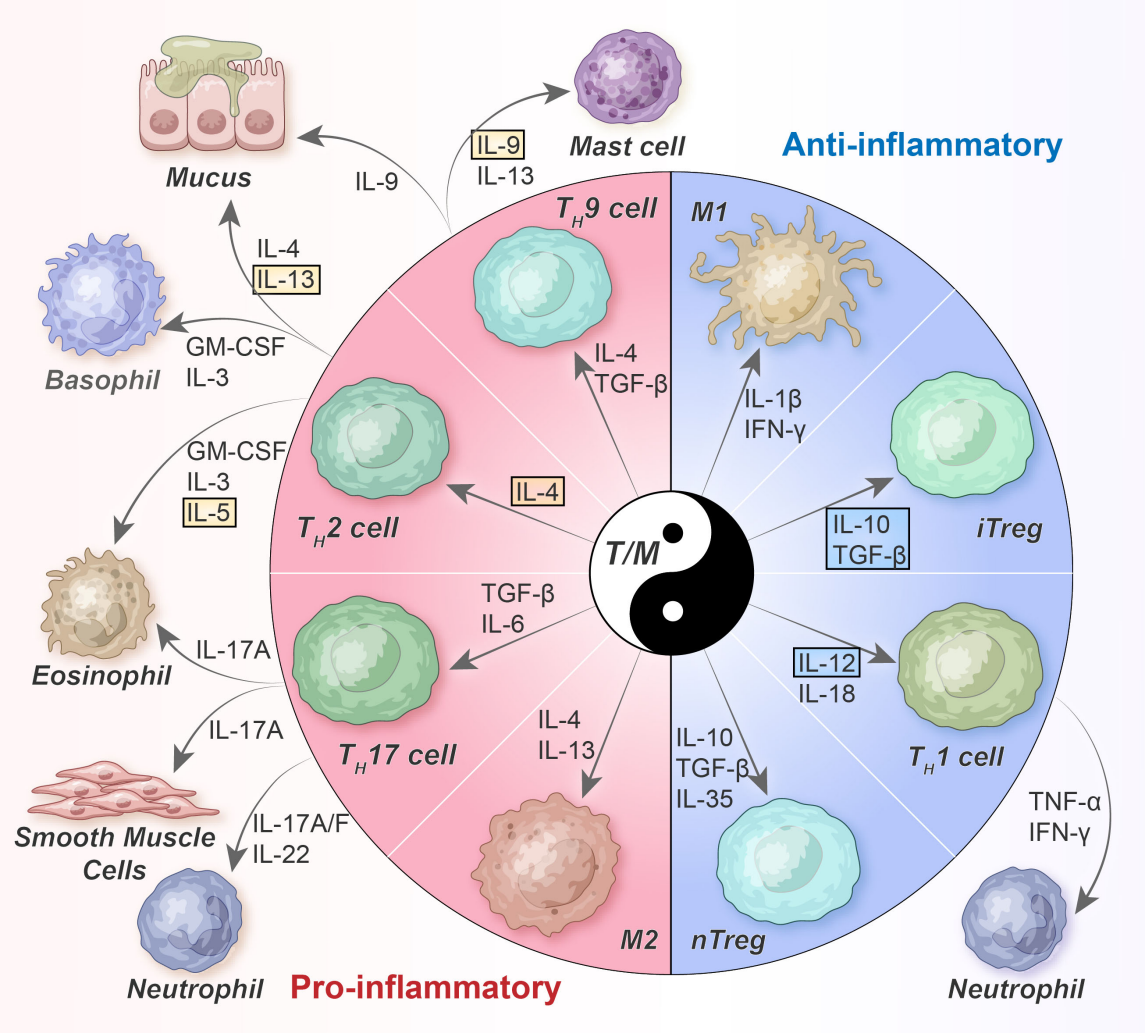
Balanced T-cell/macrophage networks and their cytokine milieu in asthma. T_H_2 cells orchestrate allergic inflammation by secreting T_H_2 cytokines such as IL-4, IL-5, IL-9, and IL-13. However, T_H_1 cells, which are differentiated because of IL-12 and IL-18, inhibit T_H_2 cells by producing IFN-γ. T_H_17 cells, affected by IL-6 and TGF-β, follow a distinct differentiation pathway. In general, Tregs mitigate the activity of other T_H_ cells by secreting TGF-β and IL-10; however, their functionality may be compromised under asthma conditions. nTreg, naturally occurring Tregs; iTreg, inducible Tregs; GM-CSF, granulocyte-macrophage colony stimulating factor; TGF, transforming growth factor; TNF, tumor necrosis factor; IFN, interferon.

Despite the well-known heterogeneity of asthma, an imbalanced immune microenvironment is a prerequisite for its development. This imbalance encompasses the dynamic interplay of T cells and macrophages, beginning from the initial stages and continuing until disease progression. Instead of attributing the disease solely to specific subsets, alterations in the interactions and functions between different subgroups may play significant roles (50). Such interactions and functions may be referred to as “pro-inflammatory/anti-inflammatory balance regulatory networks” (**Figure 1**). Notably, the classical paradigm of T_H_2-skewed immune responses remains relevant; however, emerging evidence suggests that it is considerably more sophisticated *in vivo* than previously envisaged, involving extremely uneven cell subpopulations and different cytokine expression patterns that dynamically fine-tune themselves based on different spatiotemporal cues.

## 3 Mechanisms leading to asthma

### 3.1 Step 1. Dysregulated epithelial barrier and early innate immune response

#### 3.1.1 Epithelial injury, activation, and derived signals

Bronchial epithelial cells, which are strategically positioned at the host and environment interface, play vital roles in preserving respiratory mucosal integrity and stability because of mechanical-physical barriers, ciliary clearance, and immunoregulatory functions; they serve as the first line of defense against pathogens and airborne allergens (51). Early investigation of airway asthma pathology has revealed that epithelial cells are damaged, disintegrated, and dysfunctional. As a result, the “epithelial barrier hypothesis” has been established, suggesting that various allergic and autoimmune diseases have similar triggering mechanisms (52).

Different asthma phenotypes exhibit airway epithelial abnormalities, primarily manifesting as increased epithelial leakage, decreased inflammatory thresholds, ciliated cell shedding, detached columnar cells (Creola bodies), and impaired intercellular adhesion (53). Under homeostatic conditions, an impermeable epithelial barrier is formed; this barrier is maintained primarily by tight junctions (TJs) at the apical end of columnar cells. This barrier is further reinforced via different adhesion mechanisms in the basal and basolateral surfaces of epithelial cells, including adherens junctions (AJs) and (hemi)desmosomes (54). The bronchial biopsies of patients with asthma have revealed that zonula occludens-1 (ZO-1) and occludin, which are TJ proteins, are irregular stained, suggesting functional defects in epithelial connections (55). Compared with control subjects, the cultured airway epithelial cells of patients with asthma also suggest TJ protein degradations (55); in contrast, E-cadherin and α-catenin, AJ proteins, expression is decreased (56).

The vulnerability of the airway epithelium to environmental irritants and the dysfunctional repair mechanisms after such injuries are vital for asthma development (57). Aeroallergens such as house dust mites (HDMs), pollens, and fungi, microbes such as viruses and bacteria, and environmental pollutants such as cigarette smoke, particulate matter (PM)_2.5_, and diesel exhaust, directly impede the integrity of TJ barriers of the airway epithelium (58). Furthermore, insufficient antioxidant and antiviral mechanisms in asthmatic airways may increase the susceptibility of epithelial cells to oxidative and virus-induced damage.

Genome-wide association studies (GWAS) have confirmed that the genetic predisposition for asthma development is partially associated with barrier dysfunction; single-nucleotide polymorphisms (SNPs) have been identified in multiple genes, including protocadherin-1 (*PCDH1*), cadherin-related family member-3 (*CDHR3*), and orosomucoid-like protein isoform-3 (*ORMDL3*) (53). Furthermore, experimental mouse models that simulate three asthma phenotypes, i.e., type 2^high^-eosinophilic, type 2^low^-neutrophilic, and mixed granulocytic, were employed to differentiate the effects of phenotypes on the disruption of the epithelial barrier by focusing on TJ proteins and mucins. Many TJ proteins, including ZO-1 and claudin-18, were decreased in the asthma phenotypes; however, the degree of reduction was different. In contrast, claudin-4 is only overexpressed in neutrophilic asthma. Moreover, phenotype-specific discrepancies are found in mucins: MUC5AC and MUC5B are overexpressed in the asthma phenotypes; however, it is more pronounced in neutrophilic and mixed asthma (59).

**Figure 2:**
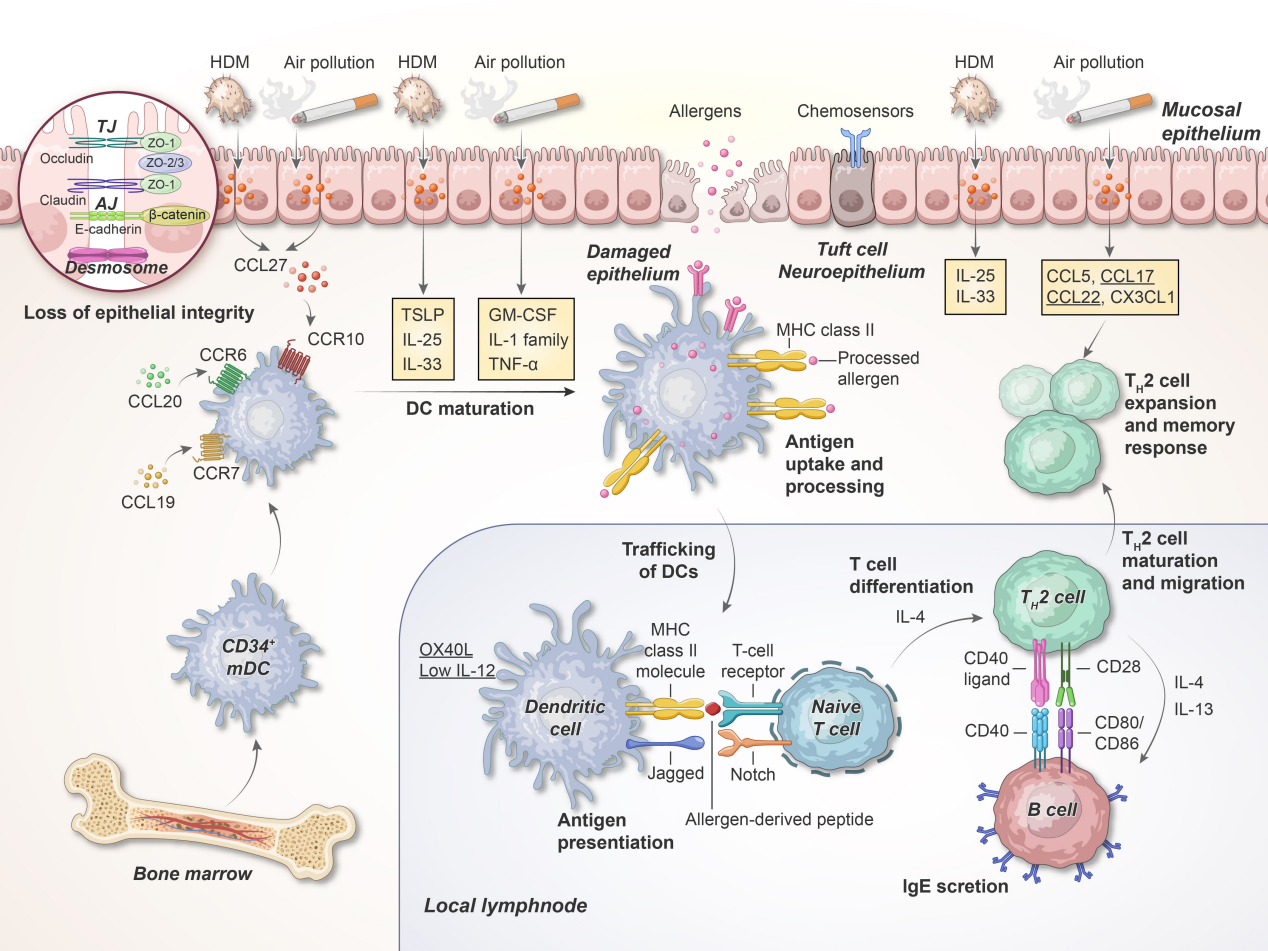
Early phase of allergen sensitizations in the airway. Inhaled allergens and air pollutants with protease activity can cleave epithelial TJs and trigger the PRRs on the epithelial cells; this results in the production of cytokines, including IL-1, IL-25, IL-33, TSLP, GM-CSF, and TNF-α. When these cytokines are released, DCs migrate toward the T cell region of the adjacent lymph nodes. Here, DCs interact with naive T cells via the TCR, MHC class II molecules, and co-stimulatory molecules, thereby facilitating T_H_ cell differentiation. HDM, house dust mite; TJ, tight junction; AJ, adherens junction; ZO, zonula occludens; TSLP, thymic stromal lymphopoietin; mDC, myeloid DCs, MHC, major histocompatibility complex. This illustration was adapted from Holgate, 2012 (75).

In addition to its essential role as a physical barrier, the airway epithelium also modulates the initial innate immune response (**Figure 2**). Epithelial cells express several pattern recognition receptors (PRRs), rapidly detecting and responding to pathogen-associated molecular patterns (PAMPs) found in microorganisms as well as damage-associated molecular patterns (DAMPs) released due to tissue injury, cellular stress and cell death (60).

Because of PRR activation on epithelial cells, large amounts of cytokines, chemokines, and antimicrobial peptides are secreted, attracting and activating innate and adaptive immune cells. During bacterial pathogen invasion, bacterial cell wall components can be sensed via various PRRs on airway epithelial cells, including toll-like receptor (TLR)-2, which recognizes elements such as lipoteichoic acid in gram-positive bacteria, TLR4, which recognizes lipopolysaccharide (LPS) in gram-negative bacteria, and NOD1 and NOD2, which recognize peptidoglycans. As a result, nuclear factor kappa B (NF-κB) is activated, initiating immune responses and eventually regulating bacterial clearance (61).

During viral infection, TLR3, TLR7/8, retinoic acid-inducible gene I (RIG-I), melanoma differentiation-associated protein-5 (MDA5), and laboratory of genetic and physiology-2 (LGP2) can recognize nucleic acid patterns (62). Furthermore, the activation of PRR-dependent epithelial cells results in the production of endogenous danger signals (i.e., DAMPs), including free adenosine triphosphate (ATP), uric acid, and high-motility group box 1 (HMGB1) protein, and activation of DCs with granulocyte-macrophage colony-stimulating factor (GM-CSF), IL-1α, and IL-33 (63).

The contemporary viewpoint suggests that tissue disturbances are the primary factor responsible for type 2 immunity rather than direct antigen recognition. In genetically susceptible individuals, with impaired epithelial barrier function, airways are vulnerable to viral infections and inhaled allergens in early life. This triggers immature DCs, guiding T_H_2 cell responses and sensitization to local allergens (64). Airway exposure to allergens, pollutants, or pathogens results in the release of epithelial-derived cytokines such as IL-33 and IL-25 and thymic stromal lymphopoietin (TSLP), a member of the IL-2 cytokine family. These cytokines, commonly called “alarmins,” exert pleiotropic properties; however, they synergistically activate DCs, ILC2s, memory T_H_2 cells, eosinophils, and mast cells, acting upstream in a sustained type 2 immune response cascade (65).

In mice, IL-33 and IL-25 activate OX40-ligand (OX40L, or CD252) expression on ILC2s, thereby activating ILC2 proliferation and cytokine production (66); in contrast, TSLP can prime DCs to improve type 2 immunity by activating T and B cells. If either one or a combination of these “alarmins” are neutralized, the development of the salient characteristics of asthma, including eosinophilia, airway hyperactivity, and peribronchial collagen deposition, may be inhibited, contingent upon the model allergen used (67–70). Moreover, clinical research suggests that after allergen inhalation for 24 h, the expression level of IL-33, IL-25, and TSLP is increased in the airway epithelium of patients with allergic asthma, correlating with the degree of airway obstruction (71). Large-scale GWAS have confirmed that SNPs in *IL33* (located at 9p24.1), *IL1RL1* (encoding the IL-33 receptor; also called suppression of tumorigenicity 2 [ST2], located at the 2q12.1 locus), and *TSLP* (5q22.1 locus) are positively associated with the risk of developing asthma; furthermore, epigenome analysis has revealed that they frequently exhibit an active chromatin state (72). Therefore, epithelial-derived alarmins may function as “early signals” in patients with different asthma phenotypes and can serve as potential therapeutic targets for allergic airway inflammation. At present, a mAb directed against TSLP (tezepelumab), has been approved for treating severe asthma in step 5 of the GINA guidelines (73). Biologic agents targeting IL-33 such as itepekimab are undergoing clinical trials (74) (**Table 1**).

**Table 1:** Classic and novel treatments developed for asthma.

Pharmacotherapies	Mechanism of action	Drugs
Traditional drugs		
Glucocorticoids	Inhibit a variety of inflammatory genes, including cytokines, inflammatory enzymes, adhesion molecules and inflammatory mediator receptors	Beclomethasone, budesonide (BUD), triamcinolone (TAA), fluticasone propionate, flunisolide (FNS)
Short/long-acting β_2_-adrenoceptor agonists (SABA/LABA)	Relax bronchial smooth muscles	Salbutamol [1968], terbutaline, formoterol, salmeterol, indacaterol
Short/long-acting muscarinic antagonists (SAMA/LAMA)	Relax bronchial smooth muscles	Ipratropium, oxitropium
Theophylline	Suppresses phosphodiesterase (PDE) and increase the concentration of cyclic adenosine monophosphate (cAMP) in smooth muscle cells	Aminophylline, diprophylline, cholinophylline, doxofylline
H_1_-antihistamine	Serves as neutral receptor antagonists or inverse agonists of the histamine H_1_ receptor, can block the action of histamine	Chlorpheniramine, loratadine, cetirizine, ketotifen
Mast cell stabilizer	Inhibits the degranulation of allergic mediators	Disodium cromoglycate, tranilast
Leukotriene (LT) receptor antagonists	Block the cysteinyl LT (cysLT) receptor type I	Zafirlukast, montelukast [1998], pranlukast
PDE4 inhibitors	Inhibit the activity of PDE4, a specific cAMP hydrolase, thus increasing the level of cAMP in cells	Roflumilast
Biologics		
Anti-IgE antibody	Binds free IgE	**Omalizumab** (FDA[2003]-, EMA[2005]-, and NMPA[2017]-approved)
Anti-IL-4R antibody	Fully humanized IgG4 monoclonal antibody (mAb) that targets IL-4Rα subunits	**Dupilumab** (EMA[2017]-, FDA[2018]-, and NMPA[2023]-approved)
Anti-IL-5 antibody	IgG1 antibody against IL-5	**Mepolizumab** (FDA[2015]-, EMA[2015]-, and NMPA[2024]- approved)
Anti-IL-5 antibody	IgG4 antibody against IL-5	**Reslizumab** (FDA[2016]- and EMA[2016]-approved)
Anti-IL-5R antibody	Inhibits binding of IL-5 to IL-5Rα	**Benralizumab** (FDA[2017]-, EMA[2018]-, and NMPA[2024]-approved)
Anti-IL-9 antibody	Blocks IL-9	MEDI-528
Anti-IL-13 antibody	Inhibits the dimerization of IL-13Rα1 and IL-4Rα	Lebrikizumab
Anti-IL-13 antibody	IgG4 mAb targeting IL-13	Tralokinumab
Anti-IL-17 antibody	Blocks IL-17	Secukinumab
Anti-IL-17R antibody	Anti-IL-17RA mAb, which blocks IL-17A, IL-17F, and IL-17E (IL-25)	Brodalumab (AMG 827)
Anti-TSLP antibody	Human IgG2 mAb against TSLP	**Tezepelumab** (FDA[2021]-approved)
Anti-IL-33 antibody	Humanized IgG4 mAb with anti-alarmin activity against IL-33	Itepekimab
Anti-ST2 antibody	Fully human IgG2 mAb that binds to ST2 and inhibits IL-33 signaling	Astegolimab
Anti-DP2 antagonist	Highly selective prostaglandin D_2_ (PGD_2_) receptor 2 (DP2) antagonists	Fevipiprant
Anti-IL-6 antibody	Humanized IL-6 receptor blocker	Tocilizumab

FDA, U. S. Food and Drug Administration; EMA, European Medicines Agency; NMPA, National Medical Products Administration of P. R. China.

#### 3.1.2 DCs deliver immunogenic messages to naive T cells

The interaction between specialized antigen-presenting airway DCs and T cells facilitates allergen sensitization. Allergen processing into small peptides and selectively presenting these processed peptides to the T cell receptors (TCRs) of naive T cells via major histocompatibility complex (MHC) class II molecules (i.e., the “first signal” or antigen-specific signal) are the underlying mechanisms (75).

Effective allergen signaling warrants co-stimulatory interplay between DCs and T cells that occurs in local lymphoid collections; this results in the differentiation of T cells into T_H_2-type T cells (i.e., the “second signal” or co-stimulatory signal) (76). Specifically, the activation of epithelial cells can result in the release of chemoattractants (C-C motif chemokine ligand [CCL]-20, CCL19 and CCL27, the ligands for CCR6, CCR7, and CCR10, respectively) that attract immature DCs that then differentiate and activate inflammation and adaptive immunity. A subset of conventional DCs (cDC2s) that depend on IRF4 for their development is responsible for initiating T_H_2 responses in mouse lungs and other organs (77). Several epithelial-derived cytokines, including IL-1α (78), GM-CSF (78), IL-33 (79), TSLP (80), and CSF1 (81), can directly target CD11b^+^CD172a (SIRPα)^+^ cDC2s, involved in the differentiation of T_H_2 cells. However, T_H_2 responses are not induced by lung-resident CD103^+^XCR1^+^ cDC1s (IRF8 and the basic leucine zipper transcriptional factor ATF-like 3 [Batf3]-dependent) and monocyte-derived DCs (moDCs). In fact, they may confer protection against asthma development by producing IL-12, a T_H_1-associated cytokine, thereby inhibiting T_H_2 responses (82). Moreover, plasmacytoid DCs (pDCs) play a tolerogenic role in allergic lung inflammation, inducing Foxp3^+^ Tregs in response to inhaled antigens, at least partially by upregulating programmed death-ligand 1 (PD-L1, or CD274), a T cell inhibitory ligand (83).

T cell differentiation is driven by the migration of allergen-loaded cDC2s to the regional lymph nodes from the lung tissues; this may be regulated by ILC2-derived IL-13 and type I IFNs (84, 85). Furthermore, epithelial cell-derived cytokines and chemokines, including IL-25, IL-33, CCL17 (thymus- and activation-regulated chemokine, TARC), and CCL22 (macrophage-derived chemokine, MDC), affect the activation of DCs, maturation of T_H_2 cells, and their mucosal migration. In asthma models challenged with allergen, CD11b^+^ DCs may be an important source of CCL17 and CCL22 (by activating their CCR4 receptors), T_H_2 cell-attracting inflammatory chemokines, and eosinophil-selective chemokines (i.e., the “third signal” or DC-secreted cytokines) (86). In clinical settings, activated DCs considerably increase in the airways of individuals suffering from asthma and ongoing inflammation (87); the high-affinity receptor FcϵRI is expressed in their lung cDC2s express (88, 89), with increased levels of CD86 and OX40L (90).

Indeed, because DCs can perceive danger signals, process antigens, and migrate to draining lymph nodes, they occupy the intersection between innate and adaptive immunities in the lungs.

### 3.2 Step 2. Adaptive immune reaction: features of type 2-high and type 2-low inflammation

**Figure 3:**
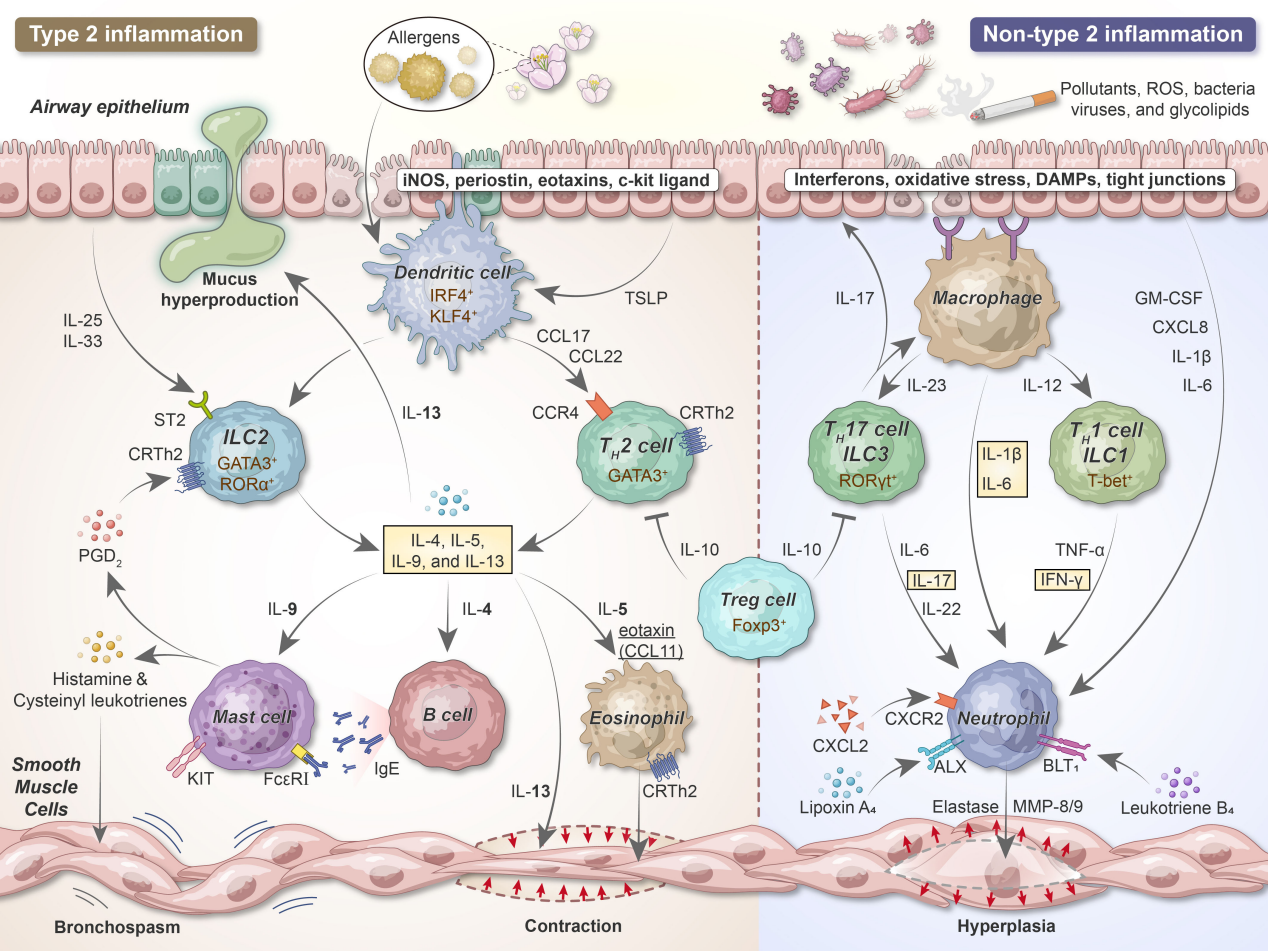
The development of airway inflammation and bronchial hyperresponsiveness in acute asthma. After sensitization, epithelial cells release alarmins (TSLP, IL-25, and IL-33) that activate DCs and ILCs. Upon the uptake, processing, and presentation of antigens to naive T cells, DCs promote naive T cell differentiation into T_H_2 lymphocytes. ILC2s and T_H_2 secrete IL-4, IL-5, IL-9, and IL-13, exerting vital roles in type 2 inflammation. Pollutants, cigarette smoke, viruses, and bacteria can damage and stimulate the airway epithelium, thereby releasing IL-1β, IL-6, and chemokines such as IL-8 acting as neutrophil chemoattractants. DCs and macrophages recruit neutrophils and release pro-inflammatory cytokines via T_H_1/ILC1 and T_H_17/ILC3 cells. iNOS, inducible nitric oxide synthase; IRF4, interferon regulatory factor 4; KLF4, Kruppel-like factor 4; ST2, suppressor of tumorigenicity 2; GATA, GATA-binding protein; ROR, RAR-related orphan receptor; Foxp3, forkhead box protein 3; T-bet, T-box expressed in T cells; PGD_2_, prostaglandin D_2_; MMP, matrix metalloproteinase.

Because tolerance or immune regulation fails in step 1, adaptive immune inflammation develops in the lung; this comprises CD4^+^ T_H_2, T_H_1, T_H_17, ILC2, and IgE-producing B cells (**Figure 3**). T_H_2 cytokines such as IL-4, IL-5, and IL-13 primarily drive allergic inflammation. However, pro-inflammatory cytokines, including TNF-α and IL-1β, augment inflammatory responses and play a role in more severe diseases. Some cytokines, including IL-10 and IL-12, exert anti-inflammatory properties and appear to be insufficient among individuals with asthma.

Diverse asthma phenotypes are primarily driven by the complex interaction between type 1 and type 2 immune pathways (**Table 2**). In the early 1990s, some years after studies on the immune system in mice helped develop the T_H_1/T_H_2 T-lymphocyte-focused paradigm, the concept that airway inflammation in atopic asthma is associated with activated T_H_2 cells was first recognized (91). In another study, GATA3 was identified as a master transcription factor for T_H_2 cell development and cytokine production (92). Likewise, T-bet (93) and retinoic acid-related orphan receptor γt (RORγt) (94) are vital for differentiating T_H_1 and T_H_17 cells, respectively. In the last decade, solid data regarding the mechanisms underlying lineage development and the molecules associated with ILC subset functions have been obtained, wherein ILC1s, ILC2s, and ILC3s share and mirror the characteristics of CD4^+^ T_H_1, T_H_2, and T_H_17 cells (95). ILC1s, similar to T_H_1 and NK cells, generate IFN-γ upon IL-12 and IL-15 stimulation and depend on T-bet for their development. ILC2s depend on GATA3 and RORα and generate type 2 cytokines such as IL-5, IL-13, and IL-9, but little IL-4. ILC3 development requires RORγt; they respond to IL-23 and IL-1β, thereby producing IL-17 and IL-22.

**Table 2:** Asthma phenotypes and cellular mechanism of type 2-high and type 2-low inflammation.

Features		T2-“high”	T2-“low”
Clinical	Age	Early-onset, children	Late-onset, adult
	Clinical behavior	Often associated with allergic rhinitis, positive skin prick test to aeroallergens or presence of allergen-specific Ig E	Corticosteroid resistant, absent of eosinophilia
	Diagnostic criteria	Blood eosinophils **≥150/μl**, and/or FeNO ≥20 ppb, and/or sputum eosinophils ≥2%, and/or asthma is clinically allergen-driven [GINA 2024]	—
	Obesity/metabolic dysfunction	May be present	Often present
	Exacerbations	Allergen induced exacerbate	Cigarette smoke, pollution and viral induced exacerbate
	Medication sensitivity	More responsive to corticosteroids and bronchodilators	Less responsive to corticosteroids and bronchodilators
Inflammatory response	Epithelial cells	Secrete TSLP, IL-33, and IL-25	Secrete IL-1β and IL-23
	DCs	DC2 express IL-4, OX-40L, CCL17, and PGE2	DCs secreted IL-6, IL-23, and TGF-β
	NKT cells	NKT cells secreted type 2 cytokines	Monocyte and NKT cells secreted IL-8
	T_H_ cells	T_H_2 secreted IL-4, IL-5, and IL-13	T_H_1 secreted IFN-γ and TNF-α
		T_H_9 secreted IL-9	T_H_17 secreted IL-17
	ILCs	ILC2s secreted type 2 cytokines	ILC3s secreted IL-17
	B cells	IgE class-switched B cells	—
	Mast cells	Mast cells secreted proteas and PGD2	—
	Effector cells	Eosinophils secreted IL-4, IL-5, IL-13, granule proteins (MBP, EPO, ECP, EDN) and cysteinyl leukotrienes	Neutrophilic or paucigranulocytic

The ability of both CD4^+^ T cells and ILCs to rapidly release various cytokines in response to environmental stimuli such as tissue damage, pathogen invasion, or cellular stress is a fundamental characteristic. Accumulating evidence indicates that the plasticity and maintenance of the subsets of T_H_ cells and ILCs are regulated by a delicate balance between their transcription factors, which are activated by differentiation-oriented cytokines; furthermore, they are affected by epigenetic modifications due to tissue microenvironment alterations (96). With an in-depth understanding of these cell types, we can gain invaluable insight into the mechanisms underlying the development of type 2-high and type 2-low asthma phenotypes (97).

#### 3.2.1 Type 2 inflammation

Immunologists use mice to set up an ovalbumin (OVA)-induced allergic asthma model; this provides a window for elucidating the pathophysiology of the type 2-high asthma endotype and developing novel therapeutics (98). This is a pure T_H_2 response in which sensitization is achieved by intraperitoneally injecting the model antigen alum (adjuvant)-emulsified OVA and challenging with aerosolized OVA; this promotes IL-4, IL-5, IL-9, and IL-13 production and OVA-specific IgE and IgG1 synthesis.

Using this model, it was discovered that IL-4, via IL-4Rα, can promote IgE class switch recombination of B cells and plasma cell differentiation, worsen bronchial hyperreactivity, and induce the expression of adhesion molecules such as ICAM-1 (CD54) and VCAM-1 (CD106), priming the vascular endothelium for eosinophils extravasation (99). Uniquely, IL-4 is critically involved in the differentiation of naive T_H_ cells into T_H_2 cells.

IL-5 is an essential cytokine for eosinophil development, maturation, activation, proliferation, and survival (100). However, it may not exert chemotactic effects on eosinophils. CCR3 is selectively activated by eotaxin-1 (CCL11), eotaxin-2 (CCL24), and eotaxin-3 (CCL26) (101), combined with the expression of some adhesion molecules, including VCAM-1 (which is upregulated by IL-4 and IL-13) (102), facilitating the recruitment of eosinophils from the bloodstream to the lung mucosa and interstitium.

IL-13 and IL-4 have similar biological properties because both cytokines can function via the IL-4Rα chain and phosphorylate STAT6, a downstream transcription factor (103). However, the key difference is that IL-13 majorly participates in the effector phase of type 2 immune responses, thereby affecting the development of several traditional pathophysiological characteristics of asthma owing to the effects it exerts on lung structural cells, including epithelial cells (mucus-secreting goblet cell differentiation and proliferation), SMCs (smooth muscle hypertrophy induction and enhanced contractility), fibroblasts (extracellular matrix [ECM] production), and endothelial cells (vascular remodeling) (8, 104). Unlike IL-4, IL-13 plays no role in the differentiation of T cells because IL-13R is not expressed in immature T cells. This disparity may be because canonical T_H_2 cells produce high levels of IL-13 at the effector site of the lungs, whereas IL-4 is primarily produced by T follicular helper (T_FH_) cells in the lymph nodes (105). Furthermore, in airway epithelial cells, IL-13 augments the expression of inducible nitric oxide synthase (iNOS); iNOS is primarily involved in generating fractional exhaled nitric oxide (FeNO), a diagnostic biomarker to examine type 2 inflammation in the respiratory tract (106).

IL-9 is released by a subset of CD4^+^ T cells (T_H_9 cells), potentially by classical T_H_2 cells, as well as by ILC2s. IL-9 drives mast cell survival, bronchial hyperresponsiveness, mucus cell metaplasia, and airway wall remodeling in mouse models (107). Therefore, IL-4, IL-5, IL-9, and IL-13, serving as classical type 2 cytokines, share common characteristics; however, each cytokine exhibits an exclusive functional profile.

The discovery that eosinophil-rich responses could be induced in mice lacking T and B cells has piqued our interest over the past few years that ILC2s as an important player in the pathogenesis of asthma (108). ILC2s are significantly increased in the blood and in the bronchoalveolar of asthmatic patients (109). Both ILC2s and T_H_2 cells belong to the lymphoid lineage and generate similar cytokine patterns. Their functions considerably overlap in asthma, although there are some detailed differences. In the presence of IL-25 or IL-33, ILC2s can directly control the key features of type 2 asthma, including eosinophilia, bronchial hyperreactivity, and goblet cell hyperplasia, by producing IL-5, IL-9, and IL-13 (110). Furthermore, ILCs function as antigen-presenting cells that use MHC class II molecules to present antigenic epitopes and express OX40L to support CD4^+^ T lymphocyte activity (111, 112). Moreover, in local tissue settings, ILC2s interact with both DCs and T_H_2 cells in complex bidirectional crosstalk, modulating the intensity of type 2 responses to properly respond to perceived environmental threats (84, 113).

Cellular activation and inflammatory mediator release are representative characteristics of type 2-high asthma, which can be observed via mast cell degranulation and eosinophil vacuolation. The interplay between IgE and high-affinity FcϵRI on granulocytes, including mast cells and basophils, results in the initiation of cell activation and degranulation, releasing multiple preformed and newly synthesized mediators, cytokines, chemokines, and growth factors. The ready-made mediators stored in cytoplasmic granules include biogenic amines (e.g., histamine and serotonin), neutral proteases (e.g., tryptase, chymase, and carboxypeptidase A), proteoglycans (e.g., heparin and chondroitin sulfate), and some cytokines (e.g., TNF-α) and growth factors (e.g., vascular endothelial growth factor A [VEGFA]) (114). T_H_2-dependent trypsin-expression cell (MC_T_) are the main type of mast cells that contribute to mild-to-moderate allergic asthma (115). However, in more severe asthma forms, mast cells containing both trypsin and chymase (MC_TC_) become dominant; compared with MC_T_, MC_TC_ relies more on stem-cell factors (SCF, i.e., the KIT ligand) for survival (116).

Lipid-derived mediators can be secreted by FcϵRI aggregation-activated mast cells. They are responsible for arachidonic acid metabolism via the cyclooxygenase (COX) and lipoxygenase (LOX) pathways, releasing prostaglandins (PGs, particularly PGD_2_), leukotriene B4 (LTB_4_), and cysteinyl leukotrienes (CysLTs, including LTC_4_, LTD_4_, and LTE_4_) (117). Histamines, PGs, and CysLTs as potent bronchoconstrictors and can lead to bronchospasm, vasodilation, plasma leakage, mucus production, and elevated cellular recruitment in the lungs. PGD_2_ acts on type 1 PGD_2_ receptor (DP1) to contract the airway smooth muscle and CRTH2, a T_H_2 cell-expressed chemokine receptor, i.e., DP2, to chemoattract T_H_2 cells, ILC2s, and eosinophils (118). Several anti-allergic drugs targeting the abovementioned mediators, including antihistamines, leukotriene modifiers, mast cell membrane stabilizers, and DP2-receptor antagonists, have been developed to decrease type 2 inflammation in the airways. These drugs are majorly used as adjunctive therapies for patients who inadequately respond to regular treatment strategies for allergic asthma, particularly those with allergic rhinitis and allergic skin diseases.

In recent years, non-steroidal anti-inflammatory-drug (NSAID)-exacerbated respiratory disease (NERD) has attracted increasing attention (119). As a prominent severe type 2-high asthma phenotype that appears in adulthood, it is characterized by increased production of CysLTs, high prevalence of coexisting chronic rhinosinusitis with nasal polyps (CRSwNP), and hypersensitivity to aspirin, emphasizing that dysregulation of arachidonic acid metabolism likely exacerbates the worsening of upper and lower airway symptoms of asthma.

#### 3.2.2 Non-type 2 inflammation

Asthma is generally linked to increased eosinophils and T_H_2 cytokines; however, some patients present with a predominantly neutrophilic disease, also called “non-type 2” or “type 2-low” asthma; this asthma phenotype lacks T_H_2 cytokine signatures but may exhibit severe glucocorticoid resistance (13). Other features associated with severe neutrophilic asthma include advanced age, impaired lung function, decreased reversibility of bronchodilator responsiveness, microbial infections, tobacco consumption, and obesity (120). Although the molecular mechanism underlying airway neutrophilic inflammation remains unelucidated, it primarily involves the IFN-γ-mediated type 1 and IL-17-mediated type 3 immune pathways (121).

In general, PRRs (such as TLRs) activation in response to microbial infections leads to a type 1 immune response; it involves immune cells that can release IFN-γ, including CD4^+^ T_H_1 cells, type 1 ILCs (ILC1s), NK cells, and CD8^+^ cytotoxic T (T_C_1) cells (122). The development of naïve T cells in the T_H_1 or T_C_1 direction can be induced by intracellular microbes that interact with the TLRs on DCs in the presence of IL-12 and IL-18 derived from DCs and IFN-γ derived from NK cells or ILC1s. The BALF cells isolated from patients with severe type 2-low asthma and lung tissues obtained from corresponding mouse models showed increased levels of IFN-γ and decreased expression of secretory leukocyte protease inhibitor (SLPI), which correlated with high airway resistance and steroid insensitivity (123). At present, the pathogenic roles of type 1 immunity in asthma remain controversial. IFN-γ signaling may be absent in the airway epithelial cells of patients with asthma (124); this abnormality increases their vulnerability to viral infections and worsens asthma (125). In contrast, other studies have demonstrated that severe asthma episodes are associated with high IFN-γ and IL-17A expression in the airways (126, 127). Despite receiving high-dose corticosteroid treatment, CD4^+^ T cells with IFN-γ^+^ (T_H_1 cells) were higher in the BALF of patients with severe asthma than in that of patients with mild or moderate asthma (123, 126).

IL-17 cytokine family members, including IL-17A and IL-17F, mediate the type 3 immune process. Extracellular bacteria and fungi induce IL-1β and IL-23 production by myeloid DCs (mDCs); as a result, primitive CD161^+^ T cells differentiate into CD4^+^ T_H_17 or CD8^+^ T_C_17 cells and trigger ILC3s to generate cytokines (122). Furthermore, IL-17A, IL-17F, and IL-22 regulate neutrophilic influx into tissues by inducing airway epithelial and stromal cells to generate cytokines such as G-CSF, GM-CSF, and IL-6, as well as chemokines such as CXCL1, CXCL6, and CXCL8 (IL-8); this promotes neutrophil activation and migration (128). Other cells, including γδT cells, NKT cells, and granulocytes, are also known to secrete IL-17 cytokines (129). In some individuals with moderate-to-severe asthma, these cytokines are increased in the blood, sputum, and bronchial biopsies, correlating with increased disease severity (130). In mice and humans, T_H_17-related cytokines play a role in airway remodeling by inducing mucous cell metaplasia, promoting fibroblast and SMC proliferation, and directly contracting bronchial SMCs, thereby narrowing the airway (31, 131). Unfortunately, despite the relatively large amount of evidence via observational studies, a randomized clinical trial of brodalumab, an IL-17 receptor-neutralizing antibody, in patients with poorly controlled moderate-to-severe asthma failed to exhibit significant benefits in all-comers (132) (**Table 1**).

#### 3.2.3 Phenotype overlap and systemic inflammation

Owing to a strong relationship between heterogeneity and asthma, the dichotomous viewpoint of asthma categorization may be oversimplified; it only appears at the extremes of the continuous spectrum (129). Based on asthma endotype complexity, two primary inflammatory signatures involving T_H_2 or T_H_17 cells and their respective cytokines have been categorized as discrete subpopulations; however, recent evidence suggests the simultaneous occurrence of type 2 and non-type 2 immune responses in some patients (133). Most T_H_ cells display a degree of plasticity that depends on environmental factors and may be redirected toward other effector CD4^+^ cells. Interestingly, a study suggested that the T_H_2 and T_H_17 inflammatory pathways are mutually regulated in patients with asthma; the suppression of the T_H_2 pathway promotes a T_H_17 response; therefore, the dual blockade of T_H_2 and T_H_17 functionalities may be rewarding for asthma treatment (134).

Historically, IL-17 was considered to be derived from conventional T_H_17 cells; however, a novel CD4^+^ T_H_2 memory or effector cell subset that collectively expresses GATA3 and RORγt and produces T_H_2 and T_H_17 cytokines has now been identified; this subset persists as the predominant IL-17-producing T cell population during the chronic phase of asthma (135). In individuals with severe and corticoid-resistant asthma, dual-positive T_H_2/T_H_17 cells (i.e., IL-17-producing T_H_2 cells) were significantly increased in the peripheral blood and BALF (136, 137). These dual-positive T_H_2/T_H_17 cells are characterized by higher levels of IL-4 production and increased expression of MEK1 (mitogen-activated protein-extracellular signal-regulated kinase [ERK] kinase 1), mediating resistance to dexamethasone-induced cell death (136). This can partially explain why a T_H_2/T_H_17^predominant^ endotype causes more severe asthma compared with the traditional T_H_2^predominat^ and T_H_2^low^ endotype (136). Additional research has demonstrated that IL-1β, IL-6, anti-IFN-γ, and IL-21, which is a cytokine environment that stimulates T cells in asthma to differentiate into the same biphenotypic cells, promote dual-positive T_H_2/T_H_17 cell differentiation, worsening asthma (137). Similarly, under specific alterations in the inflammatory microenvironment, T_H_17 cells repolarize toward the T_H_2 profile, adjusting their cytokine expression to a T_H_2-like pattern (138).

In a recent cross-sectional study in humans, phenotype distribution was examined in patients with mild-to-severe asthma (139). Phenotype overlap is extremely common in patients with asthma (73.4%), encompassing combinations of type 2-related, non-type 2-related, and mixed type 2/non-type 2 inflammation. The last group is particularly concerning; it accounts for approximately 50% of the total number of patients and exhibits the worst clinical outcomes. The average age or onset age is younger in the type 2 group, intermediate in the mixed one, and older in the non-type 2 group and might reflect immune inflammatory status evolution, beginning from a canonical type 2 signature and progressively transforming into a more complex mixed type 2/non-type 2 signature. As individuals age, those who develop pure type 2 asthma in the early years may encounter various environmental stimuli throughout their lives, possibly triggering different pathways and altering the original type 2 predominance (140).

Asthma is being increasingly acknowledged as a systemic disease, in which inflammation is not limited to the airways but remarkably cross-communicates with other distant organs by releasing inflammatory mediators (141). In patients with asthma, increased IL-6 and IL-1β levels are markers for systemic inflammation and are associated with reduced forced expiratory volume in 1 second (FEV_1_) and elevated acute exacerbations (142, 143). The analysis of the clinical traits of patients with asthma suggested that an imbalanced diet with excessive calorie intake and the resulting metabolically related inflammation can affect both innate and adaptive defense mechanisms in the respiratory tract (144). The burden of obesity-associated inflammation may increase the risk of developing asthma. White adipose tissue secretes pro-inflammatory cytokines such as IL-1β, IL-6, TNF-α, and leptin, which may negatively impact lower airway function by increasing airway hyperreactivity (145). Interestingly, increased plasma IL-6 levels and systemic inflammation are observed in both type 2-high and type 2-low asthma; however, they are not associated with upstream type 2 inflammation, with no data for the increase in IL-6 expression in the sputum (143).

### 3.3 Step 3. Transition from airway inflammation to structural changes

#### 3.3.1 Chronicity of inflammation

Inflammation persists when humans are continuously or repeatedly exposed to viruses, bacteria, allergens, air pollutants, tobacco smoke, and/or oxidative stress; furthermore, there are many innate immune cells in the bronchial mucosa epithelium, including eosinophils, neutrophils, basophils, and monocyte–macrophage lineage cells, as well as blood-derived adaptive immune cells, including T_H_2 cells, other T cell types, and B cells. Chronic inflammation and airway remodeling simultaneously occur because of the continuous cycle of epithelial damage and repair; this results in disease chronicity, which is a characteristic of asthma. In general, the inflammatory process of asthma is primarily confined to the conducting airways. However, with disease progression to a more chronic state, inflammatory cells infiltrate the proximal of the trachea and larynx and distal of the smaller airways, periodically involving neighboring alveoli. The inflammatory responses in the small airways may primarily occur outside the airway smooth muscle, whereas submucosal inflammation is predominant in the large airways.

Microscopically, inflammation can affect all layers of the airwall in patients with chronic asthma. The epithelium does not exhibit the normal pseudostratified appearance and may be stripped, with only the basal layer remaining. The basal cells are hyperplastic, with squamous metaplasia. Furthermore, goblet cell hyperplasia is observed. Characteristically, the basement membrane of the epithelium is thickened and hyalinized. Moreover, mucous glands in the bronchial submucosa are hyperplastic. In addition, the submucosa is edematous and contains a mixed inflammatory infiltrate, with different numbers of eosinophils. Bronchial smooth muscle hyperplasia and hypertrophy are the most typical features of status asthmaticus. In all bronchial tube wall layers, eosinophil, monocyte, lymphocyte, and plasma cell infiltration is observed. In some cases of fatal asthma, mucus plugs comprising mucin glycoproteins and plasma proteins may block the airways; furthermore, Charcot–Leyden crystals, the disintegrating product of eosinophils, are often observed in the tube walls and mucus plugs (146).

**Figure 4:**
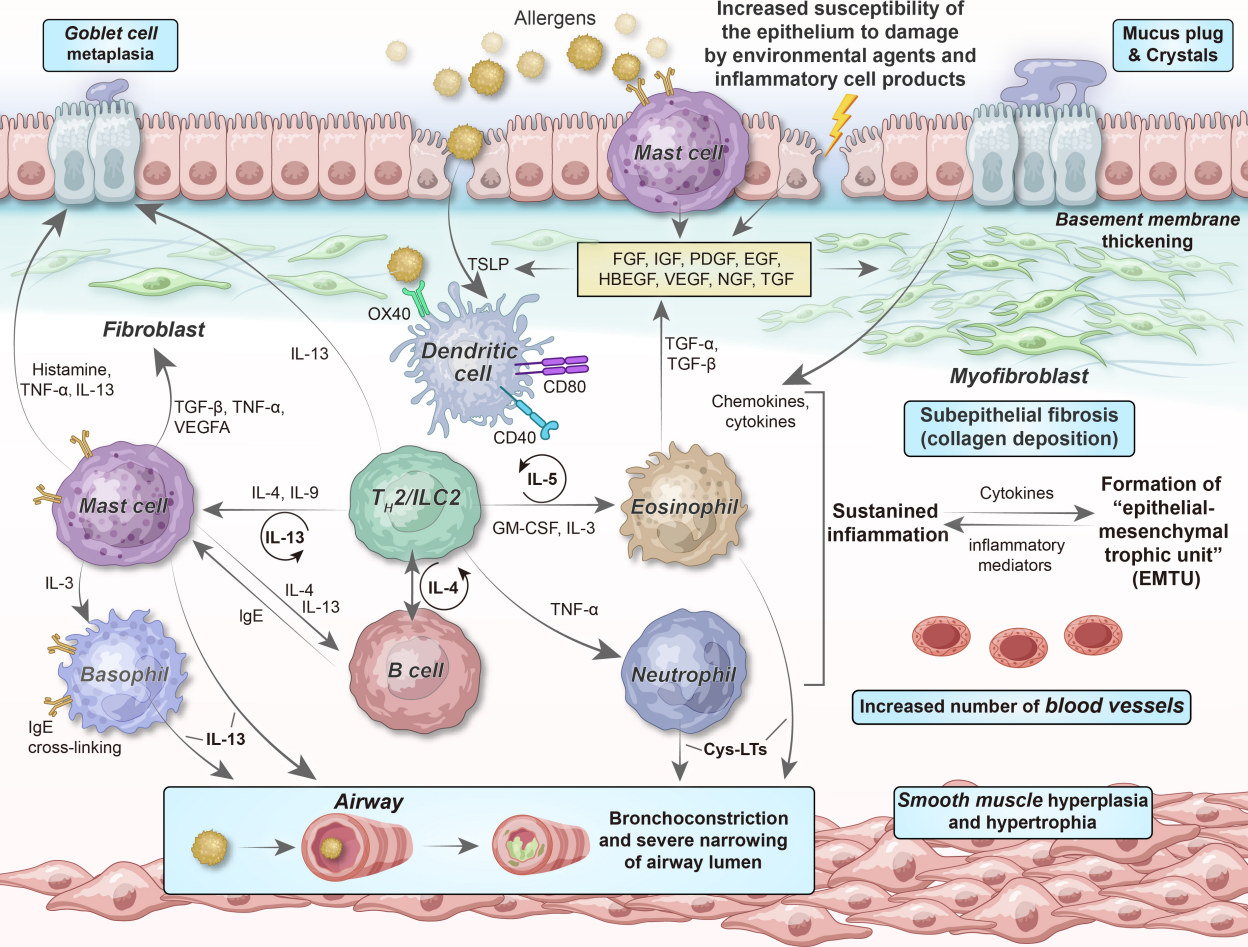
Relationship of epithelial–mesenchymal communication to airway inflammation and remodeling in chronic persistent asthma. The chronicity of inflammation is associated with repeated damage–repair responses that contribute to establishing the EMTU, which, in turn, provides a continuous tissue environment (“soil”) for T_H_2 cell/ILC2-related inflammation (“seed”). The interdependency among chronic inflammation, altered immunity, and structural changes, involving epithelial cells, (myo)fibroblasts, SMCs, and their secretory ECM, microvasculature, and neural networks, can describe why chronic airway inflammation persists even if obvious environmental stimuli are absent, as well as the reason for the failure to obtain complete therapeutic responses to anti-inflammatory drugs at the more severe and chronic end of the asthma spectrum. FGF, fibroblast growth factor; IGF, insulin-like growth factor; PDGF, platelet-derived growth factor; EGF, epidermal growth factor; HBEGF, heparin-binding EGF-like growth factor; VEGF, vascular endothelial growth factor; NGF, nerve growth factor; TGF, transforming growth factor; Cys-LTs, cysteinyl leukotrienes.

Of note, several reinforcing feedback loops promote the perpetuation of chronic airway inflammation among patients with asthma (**Figure 4**). For example, in type 2-high asthma, T_H_2 cells and ILC2s are significant IL-4, IL-5, and IL-13 sources; furthermore, infiltrating mast cells, eosinophils, and basophils play vital roles in producing type 2 cytokines. Collectively, these cells maintain the environment needed for the survival of type 2 cytokines, thereby supporting chronic disease development. IL-4 neutralizes the suppressive function of Tregs and inhibits their generation, allowing the differentiation of IL-4-producing T_H_2 cells or ILC2s (147, 148). The co-engagement of sIgE bound to FcϵRI receptors on mast cells and basophils results in the massive release of IL-4, a major mediator of B cell class switching and IgE synthesis. IL-5 mediates eosinophil differentiation and function, whereas eosinophils, in turn, secrete large amounts of IL-5. Alarmins that activate T_H_2 cells and ILC2s are released by damaged airway epithelial cells, leading to cytokine (IL-4 and IL-13) production; this significantly increase the expression of histone deacetylases (HDACs) and silent information regulator genes (SIRTs), whose activities are inversely correlated with the integrity of the epithelial barrier (149).

To break the abovementioned vicious cycle and inhibit continuous asthma progression, several anti-inflammatory drugs have been developed (**Table 1**). However, the redundancy of cytokine sources and overlapping effects of inflammatory mediators increases the robustness of the pro-inflammatory network, making treatment with a single targeted therapy (e.g., cytokine-specific monoclonal antibodies) challenging. To date, the inhaled administration of glucocorticoids remains the most effective treatment modality for asthma; they modulate (mostly downregulate) the expression of approximately 200 genes to exert anti-inflammatory effects.

Recently, the concept of “trained innate immunity” has garnered attention to explain the pathological mechanisms underlying chronic inflammation in asthma (150). Immunologic memory, long considered a specific feature of adaptive immunity, primarily depends on antigen receptor gene rearrangements and lymphocyte clone production. However, emerging literature suggests that the innate immune system can exhibit memory features (151). Abnormal innate immune memory frequently exacerbates the inflammatory responses observed in asthma. Furthermore, repeated exposure to different non-specific stimuli can result in conditionally trained immunity in innate immune cells, including airway epithelial cells, DCs, ILC2s, mast cells, monocytes, macrophages, and NK cells. Depending on the upregulation of pro-inflammatory factors such as IL-1, IL-6, TNF-α, and CCL17 or anti-inflammatory mediators such as IL-10, these cells develop into a pro-inflammatory or anti-inflammatory state. Signaling pathways impinging on transcription factors and an intricate interconnection between epigenetic modifications and metabolic reprogramming may help maintain this state, possibly favoring (pro-inflammatory state) or preventing (anti-inflammatory state) asthma development or progression (152).

#### 3.3.2 Bronchial epithelial–mesenchymal transition and lung tissue remodeling

Airway remodeling, an outstanding feature of chronic asthma, is characterized by aberrant epithelial repair and fibroblast accumulation, contributing to ECM deposition, which results in fixed bronchial obstruction (153). EMT is a dynamic approach in which epithelial cells acquire mesenchymal characteristics and lose their epithelial phenotype; it plays a vital role in normal development, tissue remodeling, fibrosis, and cancer progression. Based on its functional significance, EMT can be classified into three types: type I EMT presents during embryonic development, type II EMT participates in wound healing and organ fibrosis, and type III EMT is related to the metastasis of malignant tumors and transformation of tumor phenotypes. Among these types, type II EMT participates in asthmatic airway remodeling (154).

In general, epithelial injury and ciliopathies; mucosal edema; goblet cell hyperplasia; basal membrane thickening; increased blood vessel supply; increased mass of subepithelial fibroblasts, myofibroblasts, and airway SMCs; and ECM protein deposition are the pathological changes that occur during airway remodeling. These events result in excessive airway reactivity, with mucus formation and plugging extending into the small airways, resulting in airway trapping and overinflation; this ultimately results in decreased lung function in patients with chronic asthma (155).

As a hallmark of airway remodeling, subepithelial fibrosis is proportional to disease severity and duration (156). Through EMT, airway epithelial cells lose apical–basal polarity, undergo cytoskeletal changes, and lose intercellular adhesion and TJs; furthermore, the expression level of epithelial markers, including E-cadherin, is decreased and that of interstitial markers, including α-smooth muscle actin (α-SMA) and vimentin, is increased (157, 158); this results in airway epithelial cell differentiation into myofibroblasts, thereby exacerbating the degree of subepithelial fibrosis (159). Among the signaling pathways involved in EMT, the TGF-β_1_/Smad, Wnt/β-catenin, and Sonic Hedgehog signaling pathways have been extensively studied and occupy a central position (160).

The prevailing view is that inflammation is the primary driver and amplifier of most airway remodeling processes. Various cytokines, chemokines, and growth factors released from inflammatory and tissue cells in the airways form a complex signaling milieu, driving structural changes in the lung tissue. In type 2-high asthma, activated eosinophils release cytotoxic granular proteins, including eosinophil cationic protein (ECP), major binding protein (MBP), eosinophil peroxidase (EPO), and eosinophil-derived neurotoxin (EDN), LTC_4_, and platelet-activating factor (PAF), leading to airway constriction, mucus secretion, and increased blood permeability (161). Furthermore, eosinophils are a major source of TGF-β, inducing subepithelial fibrosis, airway smooth muscle hypertrophy, and goblet cell proliferation (162). Epithelial damage and delayed repair stimulate the production of several growth factors, including epidermal growth factor (EGF), TGF-β, and VEGF, which driving airway fibrosis and SMC, neuronal, and capillary proliferation, resembling a chronic wound scenario (163). SMCs are the primary structural cells present in the bronchial airways. During asthmatic airway inflammation, airway SMCs undergo continuous proliferation and hypertrophy, along with deposition of ECM and differentiation of goblet cells (164). Based on these structural changes, SMCs also participate in inflammatory and remodeling processes via the expression of cell adhesion molecules (CAMs), cytokine receptors, chemokine receptors, and TLRs (165). Furthermore, by releasing cytokines such as IL-4, IL-9, and IL-13, T_H_2 cells and ILC2s promote subepithelial fibrosis, epithelial goblet cell metaplasia, and SMC proliferation (166). In another striking animal study, the researchers reported that when airway inflammation levels were almost similar, mice without T_H_17 cells had lesser airway remodeling than controls. Therefore, T_H_17 cells induce airway remodeling in a T_H_2 response-independent manner (30).

Although airway remodeling is frequently associated with inflammation, this perspective is being challenged and should not be assumed to occur downstream from a single (or central) mechanism. First, airway remodeling can occur in early disease stages or preschool-aged children; however, modeling is less pronounced in adult-onset asthmatic airways (167), suggesting that it is simply not a consequence of inflammation. Second, more severe tissue remodeling, characterized by the excessive deposition of connective tissues with gradual lung function loss, is rare in moderate-to-severe asthma and only develops over time in patients receiving insufficient therapy (54). Lastly, anti-inflammatory treatment may partially reverse airway remodeling and airflow obstruction; however, it generally requires long-term rehabilitation (168).

A new hypothesis about persistent asthma has emerged in which epithelial damage in individuals with asthma triggers abnormal communication between the epithelium and basal myofibroblast sheaths, which is mediated via growth factors, thereby driving airway remodeling; this is called activation of the “epithelial–mesenchymal trophic unit (EMTU)” (169). In contrast, the cytokine milieu generated by the EMTU endows a favorable environment for sustained chronic inflammation. This leads to several functionally significant alterations in the architecture of the affected tissue, including considerable airway wall thickening (including the epithelium, lamina reticularis, submucosa, and smooth muscles), ECM protein deposition (including periostin (170), fibronectin (171), tenascin-C, osteopontin, and “repair-type” collagens I, III, V, and VI), and goblet cell hyperplasia; this is linked to increased mucus production. In individuals who have such airway wall thickening, bronchoconstriction severely narrows the airway lumen compared with that occurring in normal thickness airway walls.

Irrespective of the underlying mechanism, repeated bronchoconstriction, in and of itself, upregulates pro-fibrogenic cytokines and deposits subepithelial collagen (172). Therefore, in addition to chronic inflammation and tissue injury response, changes in mechanical stress may also lead to airway wall remodeling via the action of epithelial-inducible proteins such as YKL-40 (encoded by the gene *CHI3L1*; i.e., chitinase-3-like protein 1) (173), resistin-like molecule-β (RELMβ) (174), and members of the plasminogen activator system (175).

A groundbreaking endoscopic treatment has highlighted the importance of airway remodeling and mechanical transduction as drivers of asthma pathogenesis. Bronchial thermoplasty (BT) is a non-pharmacological intervention in which therapeutic radio-frequency energy is applied in a controlled manner to the bronchial walls to heat the tissues; BT can decrease the frequency and severity of asthmatic attacks (Evidence B) (176). This temperature-controlled radio-frequency energy is locally delivered to the proximal airways to produce clinical effects and alleviate patient symptoms by decreasing the mass of smooth muscles. However, the other effects of BT on airway remodeling and how it provides clinical benefits remain unclear, potentially involving the downregulation of cytokines such as TGF-β and RANTES (CCL5) (177), inhibition of RBM (reticular basement membrane) thickness and ECM deposition (178), modulation of innervation and vascularization, and improvement of the regenerative capacity of the airway epithelium (179). Nevertheless, evidence regarding its effectiveness and long-term safety is limited, and, at present, it can only be leveraged in research trials or within the scope of national registries as an additional treatment for some adult patients with moderate-to-severe asthma.

### 3.4 Step 4. Turning point: disease exacerbation or remission

#### 3.4.1 Allergen-induced exacerbation of type 2-high asthma

As previously discussed, in stable type 2-high asthma, the airway epithelium releases TSLP, IL-25, and IL-33, differentiating DC-activated T_H_2 cells. Therefore, IL-5 and GM-CSF are secreted by these T_H_2 cells, which along with epithelial-derived eotaxins, monocyte chemotactic proteins (MCPs), and RANTES regulate eosinophil production, maturation, recruitment, and activation. Ultimately, the local degranulation of lung eosinophils damages the airway epithelium. Meanwhile, IL-9 and IL-13, as T_H_2 cytokines, induce goblet cell metaplasia (62).

**Figure 5:**
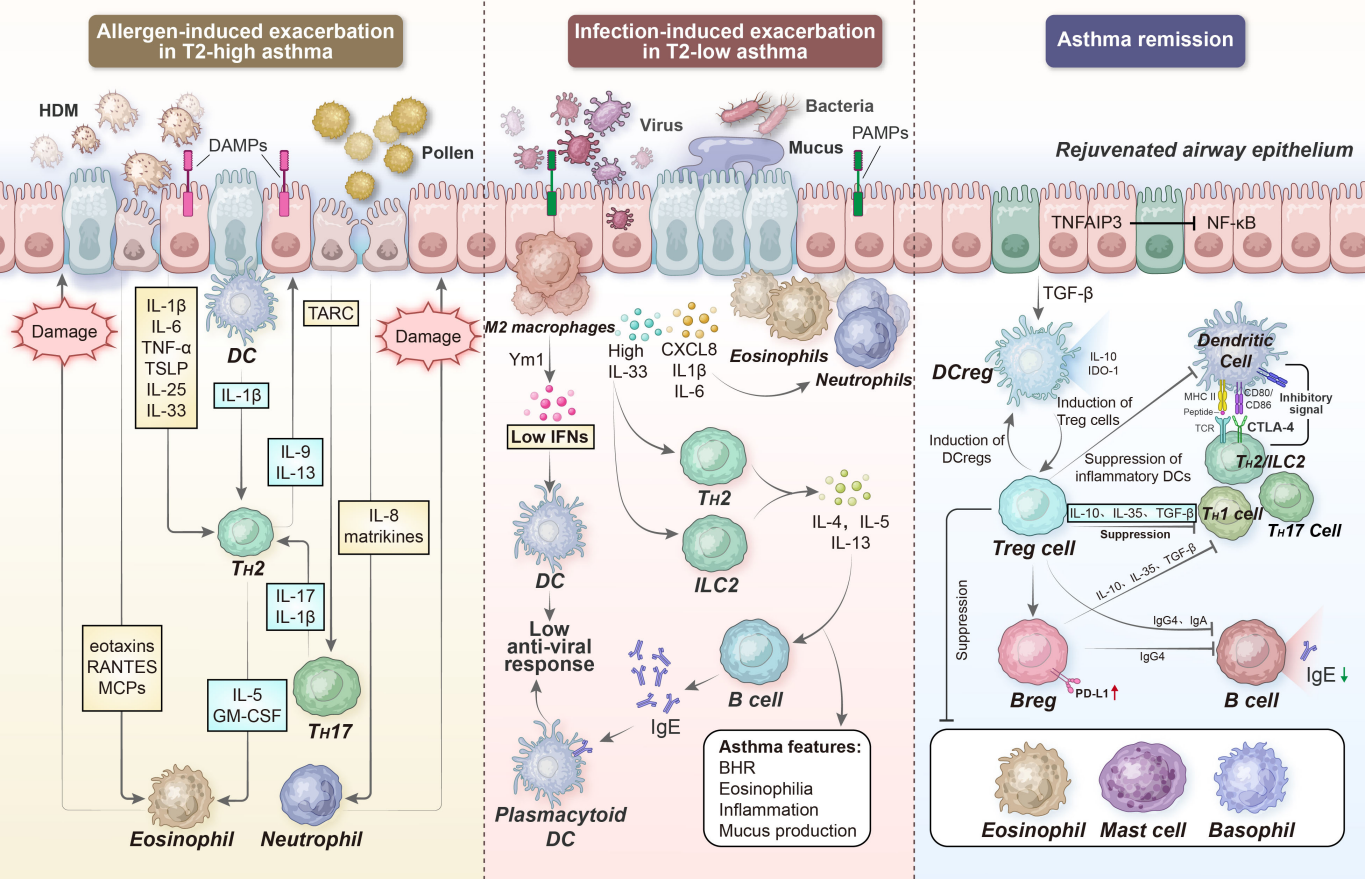
Acute exacerbation and remission of asthma. During allergen- and infection-induced asthma exacerbation, the PRRs in the airway epithelium can detect many other additional factors. Subsequently, epithelial cells in the airway release TSLP, IL-25, and IL-33 to support DC-activated T_H_2 cell differentiation; on the other hand, they secrete TARC and IL-8 to recruit T_H_17 cells and neutrophils, respectively. In turn, the local degranulation of lung eosinophils and neutrophils damages the airway epithelium, further impairing barrier integrity and enhancing ongoing inflammation. However, several regulatory immune cell types, including DCregs, Tregs, and Bregs can limit or resolve inflammation, partially via IL-10-, TGF-β-, and IL-35-dependent mechanisms. DAMPs, damage-associated molecular patterns; PAMPs, pathogen-associated molecular patterns; TARC, thymus activation regulated chemokine; RANTES, regulated upon activation normal T cell expressed and secreted; Ym1, chitinase-like protein 3; BHR, bronchial hyperresponsiveness; TNFAIP3, tumor necrosis factor alpha-induced protein 3; IDO-1, indoleamine 2,3-dioxygenase 1, CTLA-4, cytotoxic T-lymphocyte-associated protein 4.

Several other factors, including indoor allergens such as dust mites, pet fur, and cockroaches; outdoor allergens such as catkins, pollen, fungi, and cold air; and dietary allergies such as aspirin and some high-protein fish, shrimp, crabs, and eggs, can aggravate inflammatory responses during the acute worsening of type 2-high asthma. The PRRs in the airway epithelium can detect allergens and other environmental stimuli. PM_2.5_ extracts can acutely exacerbate allergic lung inflammation in an inflammasome-dependent manner via the TLR2/NF-κB/NLRP3 pathway (180). Overlaid on the general type 2-high inflammatory response, epithelial cells produce TARC (CCL17), a major chemokine for recruiting T_H_17 cells; TARC enhances the pro-inflammatory effects of T_H_2 cells by releasing IL-17 and secreting IL-8, leading to neutrophil recruitment (**Figure 5**). Evidence suggests that TARC levels are significantly elevated in the BALF of individuals with allergen-challenged asthma exacerbation. The combinations of IL-4, IL-13, and TNF-α elicit TARC release from airway SMCs, thereby promoting the force generation of smooth muscles and airway stenosis (181).

Abnormal contraction of the bronchial smooth muscle is a key pathological process that induces airway hyperresponsiveness in asthma. The inflammatory cytokines generated during asthma exacerbation, primarily IL-13, can not only directly act on IL-13 and IL-4 receptors on airway SMCs (182), thereby enhancing agonist-evoked excitatory effects by upregulating pro-inflammatory mediators such as IL-1β and TNF-α (183), but also modulate G protein-coupled receptor (GPCR, e.g., muscarinic receptor)-related signaling pathways and/or inhibit cAMP production, thereby altering calcium homeostasis in airway SMCs (184). Newly synthesized mediators released from airway SMCs promote immune cell recruitment and activation, thereby sharpening an ongoing inflammatory response. Persistent airway inflammation helps enhance the strength generated by airway smooth muscles, possibly increasing the number and size of airway SMCs. Understanding the regulation of airway smooth muscle function and combining this information with the underlying immunologic processes that drive asthma pathogenesis may become an important breakthrough in addressing the treatment mysteries of asthma (185).

In a very recent study, using a human allergen-induced asthma exacerbation model, single-cell RNA sequencing (scRNA-seq) was utilized to compare the lower airway mucosa of patients with asthma and allergic controls without asthma (186). The airway epithelium of patients with asthma was highly dynamic, with the upregulation of the genes involved in mucus metaplasia, matrix remodeling, and glycolysis; however, antioxidant and growth factor pathways observed in controls were not induced. In particular, following allergen exposure, *IL9*-expressing pathogenic T_H_2 cells were observed in the asthmatic airways, along with the enrichment of cDC2s and CCR2-expressing monocyte-derived cells, which upregulated the expression of inflammatory mediators and metalloproteinases. Moreover, a unique T_H_2-mononuclear phagocyte-basal cell interactome was discovered in patients with asthma, characterized by type 2 programming of immune and structural cells and the involvement of additional signals, including the TNF family and cellular metabolism pathways.

#### 3.4.2 Infection-induced exacerbation of type 2-low asthma

Across all age groups, respiratory tract infections are closely associated with wheezing illnesses, possibly affecting asthma development and severity. Airway infection caused by viruses, chlamydia, or mycoplasma may play a vital role in asthma pathogenesis: repeated respiratory infections during early childhood (particularly respiratory syncytial virus [RSV]) encompass the strongest predictors of future asthma risk (187); on the other hand, to date, viral infections (primarily rhinovirus [RV]) are the most common reason for the acute worsening of already established adult asthma; this results in the acute aggravation of disease symptoms, warranting increased medication use and emergency department visits and hospitalization and intensive care measures in some cases (188).

In most patients with asthma, relatively mild respiratory viruses such as RV, RSV, adenovirus, human metapneumovirus, influenza viruses, and parainfluenza viruses lead to acute exacerbation. After infection, host cells such as airway epithelial cells induce inflammatory responses to counteract the viruses. This cellular response may facilitate the acute exacerbation of asthma.

Cellular responses to viruses are initiated by TLR-3, TLR-7, and TLR-8, RIG-I, and MDA5 detecting a single-stranded RNA (or else a double-stranded RNA during replication) (189). The activation of these receptors drives a vigorous innate immune response via the induction of primary interferons (e.g., IFN-β), which results in the activation of NKT cells and alternative activation of macrophages (M2), thereby maintaining airway pathology through the production of IL-13 (190). In the lungs, anti-viral immunity heavily depends on type I (IFN-α and IFN-β) and type III (IL-29 [IFN-λ1], IL-28A [IFN-λ2], IL-28B [IFN-λ3], and IFN-λ4) IFN production, cytokine (such as IL-1, IFN-γ, TNF, IL-6, IL-12, and IL-18) secretion, and chemokine (such as CCL3, CCL5, CXCL8, and CXCL10) release (191) (**Figure 5**).

Many studies have suggested that IFN production is either delayed (192) or deficient (124, 193) in patients with asthma, resulting in the absence of adequate clearance rates for their immune responses to viral infections (194). This defect is associated with epithelial barrier and TJ disruption. Recently, studies have demonstrated that the exogenous dosing of IFN-β decreases viral exacerbations in mild-to-moderate asthma by restoring impaired antiviral innate immunity (195, 196). Additional factors that affect viral infection severity and asthma exacerbation risk include the enhanced expression of viral receptors (such as ICAM-1, low density lipoprotein receptor [LDLR], and CDHR3) (197, 198), high body mass index of patients (199), excessive increase in blood eosinophils (patients with type 2-high asthma) (199), reproducible changes in the DNA methylome (200, 201), and disturbances in the airway microbiota (202, 203).

Compared with viral respiratory infections, bacterial infections have a smaller and less important effect. In patients with acute asthma exacerbation, only a small proportion have isolated bacteria from their sputum (204).Airway bacterial infections may occur secondary to viral-induced epithelial barrier disruption, resulting in increased inflammation and risk of asthma exacerbation (205). However, there is limited evidence linking bacterial infections to acute asthma exacerbation. In a large-scale study, the colonization levels of dominant bacterial pathogens were not statistically significantly different in children with recurring and acute wheezing and those without a wheezing history (206).

Notably, the long-term oral administration of low-dose azithromycin, a macrolide antibiotic, can significantly decrease the acute exacerbation of eosinophilic and non-eosinophilic asthma, improve the quality of life of patients with severe asthma, and decrease the risk of lower respiratory tract infections (207, 208). Azithromycin exerts antibacterial, anti-inflammatory, and immunomodulatory properties and is cost-effective. At present, guidelines recommend using this antibiotic for refractory asthma. Nevertheless, potential issues such as antimicrobial resistance and side effects such as cardiac toxicity and gastrointestinal adverse reactions may restrict the widespread use of this antibiotic (209, 210).

Infection-induced asthma is not always a type 2-low inflammation. Allergic bronchopulmonary aspergillosis (ABPA) is a type 2-high inflammatory lung disease caused by an allergy to Aspergillus fumigatus, commonly presenting with treatment-resistant asthma and recurrent pulmonary shadows, which may be accompanied by bronchiectasis (211). This disease is not rare, but it is often misdiagnosed or overlooked clinically. Classical immunology suggests that the usual response of the human host to fungal clearance is a T_H_1 CD4^+^ T-cell response, which mediates the phagocytic function of macrophages and neutrophils. In contrast, the immune reaction in ABPA is predominantly mediated by T_H_2 cells, which not only cannot eradicate the fungi but also cause a massive influx and degranulation of mast cells and eosinophils, releasing various inflammatory mediators and cytokines, including IL-4, IL-5, IL-13, as well as total and *A.fumigatus*-specific IgE. Persistent inflammation will lead to airway mucus plugging, bronchiectasis, and pulmonary fibrosis. If not controlled, it can culminate in end-organ damage and clinical manifestations, which warrants our vigilance.

#### 3.4.3 Suppression and resolution of airway inflammation

Although asthma cannot be completely cured, it is possible to further pursue more realistic and achievable goals. Remission is defined as the long-term absence of disease signs and symptoms, accompanied or not by the normalization of the underlying pathology. Clinically remitted asthma requires stable lung function and the endorsement of patients or clinicians, in addition to at least 12 months without significant asthma symptoms and exacerbations; on the other hand, complete remission (cure) warrants the normalization of the underlying pathology, including resolution of airway inflammation (212). Both remission types may be achieved with or without treatment. Based on the clinical settings and research populations, clinicians and researchers can flexibly adjust the achievable and measurable definitions of asthma remission.

The introduction of ICS in the 1980s revolutionized the treatment of asthma, and to this day it remains the cornerstone for gaining optimal asthma control. ICS is generally effective in mitigating symptoms of mild-to-moderate asthma, enhancing lung function, and preventing exacerbations. The emergence of combination therapy with ICS, long-acting β_2_-agonists (LABA), and long-acting muscarinic antagonists (LAMA) has further improved asthma management. However, some studies have indicated that patients with inactive asthma, even when in complete remission, still exhibit some degree of persistent subclinical airway inflammation, hyperresponsiveness, and remodeling (213–215), possibly determining the future risk of recurrence (216, 217). Furthermore, many cross-sectional studies have examined the inflammatory markers associated with clinical and complete asthma remission in different samples such as blood, sputum, BALF, or endobronchial biopsies and eosinophils, neutrophils, mast cells, IgE, FeNO, iNOS, histamine, ECP, and EPO (214, 218–222). Most of these studies have confirmed that these markers are higher in subjects with asthma remission than in healthy controls and lower than in those with persistent asthma (223), although some studies have found no significant differences between the groups.

Further defining the clinical features and pathophysiology of asthma remission may be beneficial; however, future studies to explore its phenotype and underlying mechanisms are vital (212). Here, we focus on the immunological aspects of the natural ablation of asthma inflammation, accentuating the role of allergen immunotolerance and regulatory immune cells.

In healthy individuals, an overt immune response is not provoked during exposure to innocuous environmental antigens, if not combined with tissue damage or danger signals. Mechanistically, the clonal anergy state of T cells can be induced by isolated TCR stimulation in the absence of co-stimulatory signals. When the antigen (allergen) dose is extremely low, lymphocytes cannot be effectively activated, resulting in the unresponsiveness of the immune system (low-zone tolerance); in contrast, if the antigen over occupied TCRs, it may lead to the apoptosis of effector T cells or the induction of Treg cells that suppress immune responses, thereby also presenting an unresponsive state (high-zone tolerance). This provides a theoretical basis for the later proposed “hygiene hypothesis” and “diet-microbiome hypothesis” (224). The former suggests that in the urbanized regions of highly industrialized countries, owing to improvements in sanitation and public health and decreased exposure to infectious sources during early life, the adaptive immune system of children may not be fully exercised and developed, increasing the risk of asthma, allergic diseases, and autoimmune conditions. In contrast, rural lifestyles and large families, combined with unhygienic contact with older siblings, livestock, and soil, confer some level of protection and tolerance against allergens (225). The latter offers another explanation, i.e., a decrease in dietary fiber intake and an increase in fat intake and changes in eating habits and dietary structures will modify the composition of gut microbiota, leading to a loss of symbiotic functions of non-pathogenic bacteria and an overall reduction in microbiome diversity, as well as an increase in allergic reactions (226).

Prior exposure to some environmental factors and microbial contents, including farm dust, butyrate, LPS, and N-glycolylneuraminic acid, can suppress the responses of the airway epithelium to allergens because they induce the expression of TNF-α induced protein-3 (TNFAIP3, also called A20), a negative regulator of NF-κB activation (227). This effect inhibits the production of IL-33, GM-CSF, and the DC chemokine CCL20 by epithelial cells in response to allergen inhalation, and promotes the production of tolerance-inducing cytokines such as TGF-β; this induces tolerogenic or regulatory DCs (DCregs) that promote Treg development. In particular, the dampening of immunologic responsiveness and maintenance of allergen tolerance contribute to regulatory immune cells, notably Tregs and regulatory B cells (Bregs) (228) (**Figure 5**). Tregs exert a broad suppressive effect on several immune cells; this effect is achieved by secreting cytokines such as IL-10 and TGF-β and other inhibitory factors such as indoleamine 2,3-dioxygenase (IDO-1), as well as by providing co-inhibitory molecules such as cytotoxic T-lymphocyte-associated protein 4 (CTLA-4), programmed cell death protein 1 (PD-1, or CD279), and PD-L1 (229). Tregs directly suppress mast cells, eosinophils, basophils, ILC2s, and inflammatory DCs involved in programming effector T cell subsets (T_H_1, T_H_2, and T_H_17 cells). Furthermore, In Treg-derived cytokines, primarily IL-10, IL-35, and TGF-β, induce Bregs and produce IgG4 and IgA antibodies via B cells. In addition, Bregs produce anti-inflammatory cytokines, thereby suppressing effector T-cell responses (230).

A key event in generating normal immune responses to allergens is redirecting allergen-specific effector T cells toward a regulatory phenotype; this phenomenon accounts for some degree of the clinical efficacy of allergen-specific immunotherapy (AIT) (231). In instances where an immunologically proven allergen-driven mechanism of asthma, AIT represents the sole etiologic remedy for allergic manifestations (232, 233). It involves repeatedly administering high doses of causative allergens, generally via subcutaneous injection (SCIT) or sublingual application (SLIT), to induce a permanent state of tolerance and long-term benefits after discontinuing the treatment (234). AIT can be safely used to treat adolescents and adults with mild-to-moderate and well-controlled allergic asthma, not only significantly controlling symptoms and decreasing acute attacks but also decreasing the need for ICS dosage. Furthermore, AIT can prevent the further development of rhinitis into asthmatic symptoms in children (235). However, caution should be exercised when using AIT to treat uncontrolled and severe asthma (236).

## 4 Challenges ahead and future directions

Despite being in close contact with harmful chemicals and pathogenic microorganisms in the external environment, the human lungs can maintain efficient gas exchange. This relies on the protective role of the immune system of the lung mucosa against various harmful factors. The dynamic regulation of lung structure and immune cells is an essential guarantee for maintaining lung immune homeostasis. The disruption of this immune homeostasis may result in asthma development. As a heterogeneous condition, asthma presents with chronic airway inflammation, with the involvement of various cells and cellular components and association with airway hyperresponsiveness. If bronchial asthma is not promptly diagnosed and treated, it can lead to irreversible airway narrowing and tissue remodeling with disease progression.

In the last few years, a significant paradigm shift has been observed in our perception of asthma pathobiology, largely because of an improved understanding of its heterogeneity and different endotypes. Initially, asthma was considered a unique T_H_2 cell-mediated disease, a dogma largely developed from mouse asthma models that drove the development of several type 2-oriented monoclonal antibodies. As present, these biologics are successfully used in clinical settings, primarily to decrease the frequency of exacerbations in patients receiving conventional therapy. However, this immune process is absent in 50% of patients; this condition is termed “type 2-low asthma,” whose existence and definition remain uncertain, encompassing various asthma subtypes such as neutrophilic, mixed granulocytic, or paucigranulocytic forms and is characterized by normal eosinophils and low type 2 inflammation marker expression.

Various aspects of innate or adaptive immunity responses to allergens, environmental triggers, or viruses are involved in developing allergen sensitization, asthma symptoms, exacerbations, and treatment responses. Extensive crosstalk exists between airway epithelial and immune cells during disease initiation and persistence, indicating that epithelial barrier restoration in asthma requires greater attention. With an increase in the complexity and severity of disease manifestations, the complexity of the accompanying immunopathology also increases, with the possible involvement of additional adaptive immunity elements and structural changes in the airways. While there has been significant advancement in the understanding of some of the current immunological advances in asthma, additional studies are warranted to ascribe the mechanisms underlying asthma inception and identify additional biomarkers that facilitate targeted interventions, by prioritizing the development of tools for the rapid, accurate, and low-cost diagnosis of the endotypes and sub-endotypes of asthma.

Clinicians have begun to realize that the one-size-fits-all “stepwise approach to therapy” cannot entirely meet the optimal diagnostic and therapeutic needs of all patients, particularly those with severe or refractory asthma. The concept of “treatable traits (TTs)” has been proposed as a way to address the diverse pathophysiological factors involved in severe asthma and overcome the limitations of existing treatment strategies (237). This notion of personalized medicine, which advocates multidisciplinary teamwork and is based on multidimensional assessment, represents a shift toward precision medicine. By offering greater flexibility and comprehensiveness in treatment, this concept can significantly improve health-related quality of life and asthma control while decreasing acute exacerbations (238, 239).

Immunological advances have always resonated with the progress of clinical asthma research, mutually complementing each other. However, new curative or even preventive treatments that can control symptoms in patients with asthma are warranted in the future, to alleviate the significant burden it places on society. For example, motivated by the marked success of an adoptive cellular immunotherapy based on the chimeric antigen receptor (CAR) for treating various malignant tumor types, a research group developed a cytokine-anchored CAR-T (CCAR-T) cell system using chimeric IL-5/CD28/CD3ζ receptors and revealed the targeted killing effect of IL-5-anchored CCAR-T cells on eosinophils *in vivo* and *in vitro*, as well as their protective effect on allergic airway inflammation, significantly surpassing the quintessential therapeutic window of current mAb-based treatments in clinical settings (240). This research group innovatively employed the CCAR-T cell system to treat severe eosinophilic asthma, which may be a milestone achievement in future research on various intractable allergic diseases.

A unified and innovative approach is required to address the challenges posed by asthma. Contemporary cutting-edge methods, including but not restricted to single-cell sequencing, phenomics, genetic lineage tracing, tissue imaging systems, and organoid technology, which can achieve obtain highly multiplexed information with subcellular spatial resolution, and their in-depth computational analysis may help better define asthma in the forthcoming years. It is imperative to underscore that the key to the successful development of personalized and phenotype-specific asthma treatments lies in continuously collaborating with clinical experts and immunologists and integrating bench and bedside approaches.
